# Maize Transcription Factor *ZmARF4* Confers Phosphorus Tolerance by Promoting Root Morphological Development

**DOI:** 10.3390/ijms23042361

**Published:** 2022-02-21

**Authors:** Jing Li, Fengkai Wu, Yafeng He, Bing He, Ying Gong, Baba Salifu Yahaya, Yuxin Xie, Wubing Xie, Jie Xu, Qingjun Wang, Xuanjun Feng, Yaxi Liu, Yanli Lu

**Affiliations:** 1 State Key Laboratory of Crop Gene Exploration and Utilization in Southwest China, Sichuan Agricultural University, Wenjiang 611130, China; lijing3@stu.sicau.edu.cn (J.L.); wfk124@sicau.edu.cn (F.W.); a2668285934@163.com (Y.H.); hebingstar@163.com (B.H.); gying1719@163.com (Y.G.); bsyahayat@yahoo.com (B.S.Y.); xieyx4301@163.com (Y.X.); chuck00544@163.com (W.X.); jiexu28@gmail.com (J.X.); wdqdjm@126.com (Q.W.); xuanjunfeng@sicau.edu.cn (X.F.); liuyaxi@sicau.edu.cn (Y.L.); 2 Maize Research Institute, Sichuan Agricultural University, Wenjiang 611130, China; 3 Key Laboratory of Biology and Genetic Improvement of Maize in Southwest Region, Ministry of Agriculture, Wenjiang 611130, China; 4 Triticeae Research Institute and Key Lab for Major Crop Diseases, Sichuan Agricultural University, Wenjiang 611130, China

**Keywords:** *ZmARF4*, phosphorus stress, root morphological development, maize

## Abstract

Plant growth and development are closely related to phosphate (Pi) and auxin. However, data regarding auxin response factors (ARFs) and their response to phosphate in maize are limited. Here, we isolated *ZmARF4* in maize and dissected its biological function response to Pi stress. Overexpression of *ZmARF4* in Arabidopsis confers tolerance of Pi deficiency with better root morphology than wild-type. Overexpressed *ZmARF4* can partially restore the absence of lateral roots in mutant *arf7 arf19*. The *ZmARF4* overexpression promoted Pi remobilization and up-regulated *AtRNS1*, under Pi limitation while it down-regulated the expression of the anthocyanin biosynthesis genes *AtDFR* and *AtANS*. A continuous detection revealed higher activity of promoter in the Pi-tolerant maize P178 line than in the sensitive 9782 line under low-Pi conditions. Meanwhile, GUS activity was specifically detected in new leaves and the stele of roots in transgenic offspring. ZmARF4 was localized to the nucleus and cytoplasm of the mesophyll protoplast and interacted with ZmILL4 and ZmChc5, which mediate lateral root initiation and defense response, respectively. *ZmARF4* overexpression also conferred salinity and osmotic stress tolerance in Arabidopsis. Overall, our findings suggest that *ZmARF4*, a pleiotropic gene, modulates multiple stress signaling pathways, and thus, could be a candidate gene for engineering plants with multiple stress adaptation.

## 1. Introduction

Plants constantly encounter biotic and abiotic stress throughout their life cycles. Mahajan and Tuteja [1] suggested that these stress conditions are exacerbated by current trends in climate change. Phosphorus is one of the most important macro-nutrients that plays a pivotal role in plant growth and metabolic processes. Inorganic phosphate (Pi), the readily available form in which plants obtain their phosphorus nutrition, is prominently limited in most agricultural systems due to immobilization by complex soil chemistry [2,3,4]. Nevertheless, under Pi-limited conditions, plants have evolved to include multiple morphological and biochemical adaptations to remobilize Pi and maintain Pi homeostasis [5]. Morphological adaptations involving root system remodeling, including inhibition of primary root extension, enhancement of lateral root, and root hair proliferation [6], enhance soil scavenging, and the Pi remobilization.

Results from Pi stress response studies have provided insight into the molecular mechanisms governing Pi stress adaptation in plants. Transcription factors, such as GmWRKY45 [7], OsPHR2 [8], ZmPTF1 [9], and AtBHLH32 [10], play variable roles in Pi stress responsiveness in plants. The transcriptional response to auxin is critical for plant growth and development, including root and vascular differentiation mediated by phosphorus deficiency. Phosphate changed the distribution of auxin by modulating auxin sensitivity via the auxin receptor of TRANSPORT INHIBITOR RESPONSE 1/AUXIN SIGNALING F-BOX (TIR1/AFB) proteins [11], resulting in decreased auxin concentration in the initial region of lateral root primordia, and thus inhibited lateral root formation [12]. The nuclear auxin pathway (NAP) involves hormone perception and the subsequent alterations in auxin-responsive transcription expression mediated by the TRANSPORT INHIBITOR RESPONSE 1/AUXIN SIGNALING F-BOX (TIR1/AFB)-AUXIN/INDOLE-3-ACETIC ACID (Aux/IAA) co-repressor system and the auxin response factor (ARF) transcription factors [13,14]. Under low cellular auxin concentrations, Aux/IAA binds to ARF through their shared PB1 domain and recruits the co-repressor TOPLESS (TPL) to inhibit auxin-responsive elements (AuxREs) [15,16,17], whereas, with the increasing of auxin levels, the auxin-mediated interaction between Aux/IAA and SCF^TIR1/AFB^ leads to Aux/IAA proteasomal degradation, releasing ARFs from repression and inducing the transcriptional activity [14,18].

ARFs involved in diverse growth and developmental processes, including senescence [19], hormonal signaling [20] and developmental processes [21,22], via mediating the expression of auxin-responsive genes. The modular structure of the ARF family of proteins comprises an N-terminal DNA-binding domain (DBD), a variable middle transcription regulation domain, and a C-terminal PB1 dimerization domain [23,24]. ARF transcription factors participate in the auxin signaling pathway and bind to the TGTCTC-containing cis-regulatory AuxREs found in the promoter sequence of auxin response genes [25].

*ARF* genes have been implicated in plant responses to multiple environmental stresses [26]. The regulatory mechanism of the ARF family of proteins in multiple biological and physiological processes is a potential target to develop crop plants with superior responses and adaptability to stress. Several protein members of the ARF family have been identified and characterized in various crop species, including tomatoes [24,27], rice [28,29], and maize [30,31]. Completion of the maize genome sequence provides the most essential resource for gene discovery in maize, which could lead to the isolation and characterization of candidate genes for yield enhancement under severe environmental stress conditions through genetic engineering. In our previous research, RNA-sequencing analysis of 89 elite maize lines showed no single-nucleotide polymorphisms (SNPs) in the coding sequence of *ZmARF4* (unpublished data). Most ARFs have structures with highly conserved domains [26].

In this study, we clone the full coding sequence of *ZmARF4* and its promoter sequence from Pi-tolerant extreme maize inbred lines to evaluate the functions and expression characteristics under Pi starvation. Our results revealed that the expression level of *ZmARF4* was significantly induced by low-phosphorus stress, while the expression pattern was significantly different in the two extreme sensitivity maize inbred lines. Furthermore, overexpression of *ZmARF4* enhanced Pi stress tolerance through Pi remobilization and translocation from the roots to the leaves, and partially restored lateral root emergence and development in the *arf7 arf19* mutant. Overall, these results provide an excellent candidate gene for tolerance to a phosphorus stress response.

## 2. Results

### 2.1. Promoter Activity of ZmARF4 Was Induced by Low-Phosphorus Stress

**Figure 1:**
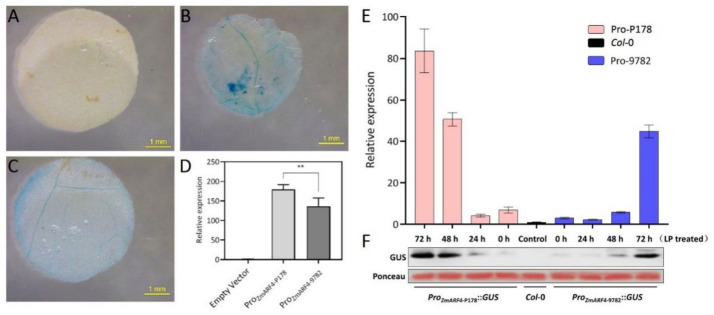
Activity detection of *ZmARF4* promoter fused with *GUS.* (**A**–**D**) GUS activity detection after transient expression in tobacco. (**A**) is the negative control; (**B**,**C**) is injected with Pro*_ZmARF4_*_-P178_::*GUS* and Pro*_ZmARF4_*_-9782_::*GUS* in tobacco leaves, respectively; (**D**) is the relative expression of *GUS* from (**A**–**C**), **, *p* < 0.01. *GUS* transcript (**E**) and protein accumulation (**F**) in transgenic lines of Arabidopsis increased with duration of LP exposure and peaked at 72 h. Pro*_ZmARF4_*_-P178_::*GUS* recorded higher relative to Pro*_ZmARF4_*_-9782_::*GUS*.

*ZmARF4* (Zm00001eb067270), as with most *ARFs* [26], encodes highly conserved protein domains. Analysis of sequence polymorphism carried out from Pi-tolerant inbred (P178) and Pi-sensitive inbred (9782) did not detect any SNPs in the coding sequence of *ZmARF4* (Figure S1). However, there are numerous SNPs existing in the promoter sequence of *ZmARF4*; among these are two inbred lines (Figure S2). In order to account for cultivar differences in phosphorus tolerance, we constructed transient expression vectors driven by different promoters of *ZmARF4* from P178 and 9782. Higher GUS activity was detected in tobacco leaves transiently expressing Pro*_ZmARF4_*_-P178_::*GUS* than in Pro*_ZmARF4_*_-9782_::*GUS* (Figure 1B,C). This was confirmed via qPCR analysis, where expression results showed higher transcript levels of Pro*_ZmARF4_*_-P178_::*GUS* compared to Pro*_ZmARF4_*_-9782_::*GUS* after transient expression in tobacco (Figure 1D).

GUS activity was also detected in transgenic plants harboring the promoter of *ZmARF4* driving the *GUS* gene. Our results showed a progressive increase in GUS activity under low-phosphate (LP) conditions with peak expression at 72 h (Figure 1E,F). GUS activity detection in Pro*_ZmARF4_*_-P178_::*GUS* was significantly higher than that in Pro*_ZmARF4_*_-9782_::*GUS* at all sampling times under low-Pi conditions (Figure 1E). This result was validated via Western blotting analysis, where GUS protein accumulation peaked at 72 h after LP treatment, with Pro*_ZmARF4_*_-P178_::*GUS*, which recorded higher GUS protein accumulation compared to Pro*_ZmARF4_*_-9782_::*GUS* (Figure 1F). GUS staining analysis in the Pro*_ZmARF4_*_-P178_ transgenic lines detected GUS stains in the stele of roots and in the lower leaves (Figure S3), suggesting that *ZmARF4* is expressed in multiple organs and plays diverse critical roles during plant development.

### 2.2. Expression Characterization of ZmARF4

**Figure 2:**
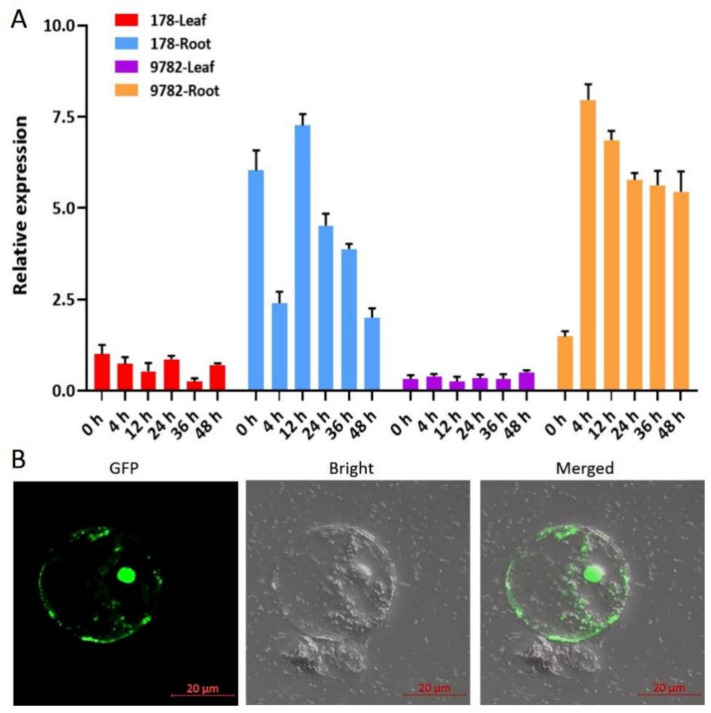
Expression characteristics of *ZmARF4*. (**A**) Tissue-specific expression of *ZmARF4* transcript from P178 and 9782 maize lines with low-Pi treated. (**B**) Sub-cellular localization of *ZmARF4* in maize mesophyll protoplast.

We used qPCR analysis to quantify the expression of *ZmARF4* in the roots and leaves of inbred lines P178 and 9782, and observed differential organ-specific expression of *ZmARF4* within and between lines (Figure 2). Transcription levels in leaves generally did not differ under normal conditions (Figure 1E); conversely, under Pi stress conditions, expression analysis in roots revealed a higher transcription of *ZmARF4* in 9782 than in P178 at all sampling times (Figure 2A). Expression in the root of 9782 peaked after 4 h of treatment and decreased gradually until 48 h. The expression level in the roots of P178 decreased after 4 h of treatment, peaked after 12 h, and then decreased gradually until 48 h (Figure 2A).

The full-length coding sequence of *ZmARF4* without the stop codon, fused with GFP under the control of the CaMV35S promoter, was constructed to determine the functional location of *ZmARF4*. The construct was transformed into maize mesophyll protoplasts and subcellular GFP expression was observed. Our results revealed that the *ZmARF4*-GFP fusion protein was localized in the nucleus and cytoplasm together (Figure 2B), suggesting that *ZmARF4* may contain some biological activities in addition to being transcriptional regulators.

### 2.3. ZmARF4 Enhanced Pi Stress Tolerance and Lateral Root Proliferation in Arabidopsis

**Figure 3:**
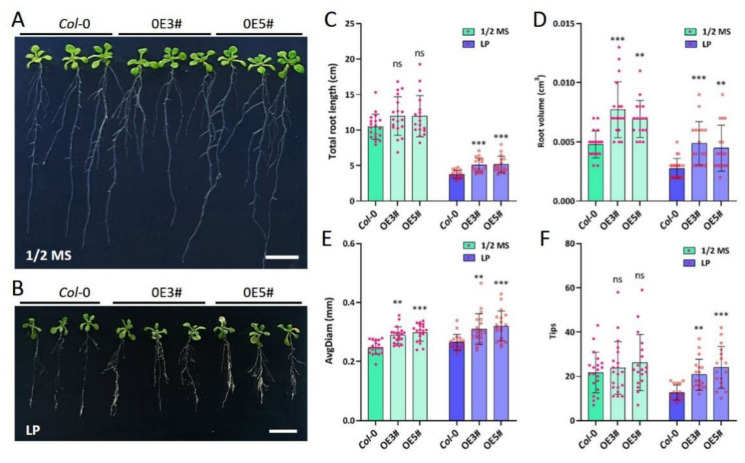
Phenotypic evaluation of transgenic Arabidopsis. Seven-day-old seedlings were transferred to (**A**) ½ MS and (**B**) low-Pi conditions for 7 days. Transgenic lines showed better tolerance to LP compared to WT and recorded significantly higher (**C**) total root length, (**D**) root volume, (**E**) root diameter, and (**F**) root tips. **, *p* < 0.01; ***, *p* < 0.001; ns, no significance.

To assess the role of *ZmARF4* in the Pi stress response, 7-day-old transgenic seedlings were exposed to low-Pi conditions for 7 days. The transgenic plants compared to the wild type (WT) showed significant differences in root volume and average root diameter under normal conditions but did not differ in total root length and tips (Figure 3A–F). Under Pi stress conditions, however, there was a significant difference in all measured root phenotypic traits; the transgenic plants had significantly higher total root length, root volume, root diameter, and tips than the WT plants (Figure 3B–F). This suggests that *ZmARF4* probably plays a pivotal role in root system architecture remodeling as an adaptive measure of Pi stress.

**Figure 4:**
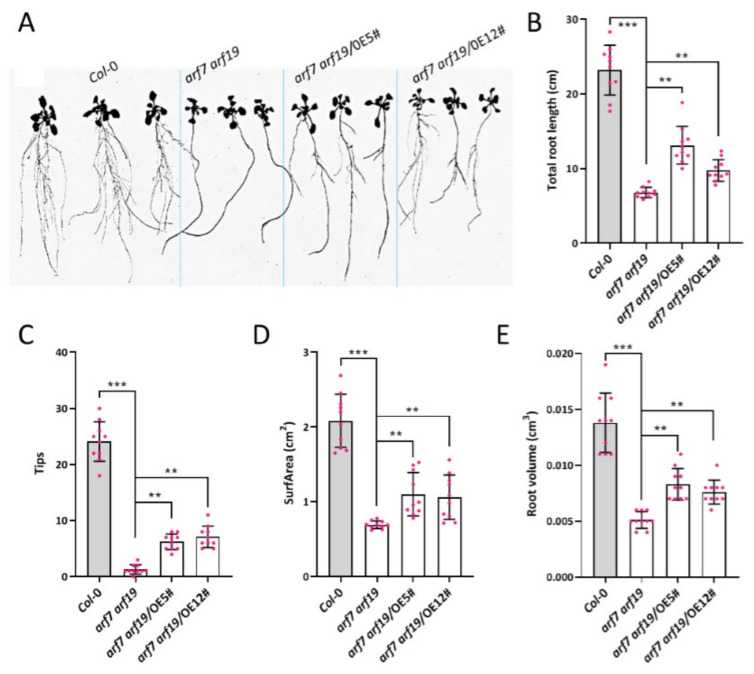
*ZmARF4* induce lateral root growth and overall root development. (**A**) Overexpression of *ZmARF4* partially restores lateral roots in *arf7 arf19* mutant. (**B**–**E**) *ZmARF4* overexpression promoted root phenotypic traits such as (**B**) total root length, (**C**) root tip, (**D**) root surface area, and (**E**) root volume. **, *p* < 0.01; ***, *p* < 0.001.

*ZmARF4* was overexpressed in the double mutant *arf7 arf19*, driven by the *35S* promoter to examine the biological function of ZmARF4 in lateral root induction. Numerous T_4_ Pro*_35S_*::*ZmARF4* lines showed variable levels of lateral root proliferation (Figure 4A). Although the number of lateral roots in Pro*_35S_*::*ZmARF4*/*arf7 arf19* was not as pronounced as in the WT, overexpression of *ZmARF4* partially restored lateral roots in the a*rf7 arf19* background, which otherwise exclusively lost lateral root emergence (Figure 4A). Consequently, Pro*_35S_*::*ZmARF4*/*arf7 arf19* lines recorded significantly higher total root length, root tips, root volume, and root surface area than *arf7 arf19* lines (Figure 4B–E).

### 2.4. ZmARF4 Improved the Tolerance of Other Abiotic Stress in Arabidopsis

To examine the possibility of *ZmARF4* in regulating other abiotic stress responses, 7-day-old transgenic seedlings were exposed to salinity and osmotic stress for 7 days. Response to these stresses was assessed by examining modifications in the root system architecture. Transgenic plants exhibited greater tolerance to salinity and osmotic stress, and recorded significantly higher total root length, tips, root volume, and root surface area compared to WT (Figure S4). This finding suggests that *ZmARF4* is a pleiotropic gene significantly involved in plant responses to multiple abiotic stress conditions.

### 2.5. ZmARF4 Increased Pi Content and Defended Anthocyanin Accumulation in Leaves

**Figure 5:**
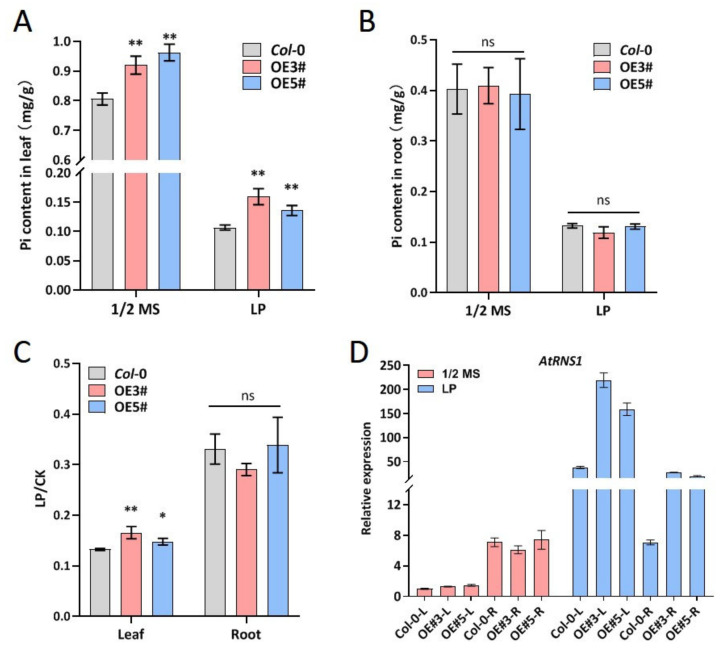
Overexpression of *ZmARF4* promotes Pi remobilization and transcription of Pi stress-responsive genes. (**A**) Pi accumulation in the leaves of transgenic lines was significantly higher than WT. (**B**) Transgenic lines and WT showed no significant difference in root Pi remobilization. (**C**) The ratio of Pi content compared after low-Pi stress in leaf and root. (**D**) AtRNS1 is differentially expressed in roots and leaves of transgenic lines and WT. *, *p* < 0.05; **, *p* < 0.01; ns, no significance.

Pi mobilization under low-Pi conditions was elucidated by quantifying Pi in the roots and leaves of transgenic lines relative to the WT. Pi content was significantly higher under normal conditions than under low-Pi conditions (Figure 5A,B); in the leaves of transgenic lines and WT plants, the Pi content was significantly different under normal and low-Pi conditions but similar in the roots (Figure 5A,B). After low-Pi stress, the ratio of Pi content was significantly higher in the leaves of transgenic plants than in WT plants but did not differ in the roots (Figure 5C). This suggests that the leaves serve as a sink for phosphates under Pi stress, such that the Pi mobilized by roots under Pi stress is translocated to the leaves.

Tissue-specific expression of the Pi stress-responsive gene A*tRNS1* [32] was also assessed. Although transcription of A*tRNS1* did not differ between the transgenic lines and the WT under normal conditions, it was relatively higher in roots than in leaves (Figure 5D). This suggests that A*tRNS1* might play a role in Pi mobilization by roots under normal conditions. The dynamics of A*tRNS1* transcription under Pi stress varied considerably, and transcription in leaves was significantly higher than that in roots, with the transgenic plants expressing higher transcripts of A*tRNS1* in leaves and roots than in the WT plants (Figure 5D). The up-regulation of A*tRNS1* in leaves of transgenic plants under low-Pi conditions suggests a possible role for A*tRNS1* in Pi translocation from the root to the leaf under Pi stress.

**Figure 6:**
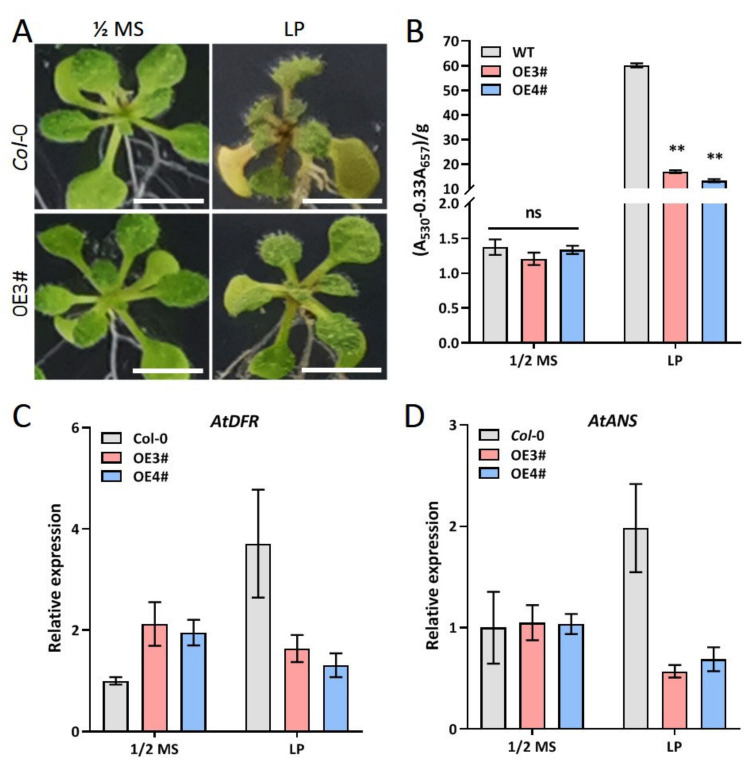
*ZmARF4* is a negative regulator of anthocyanin biosynthesis. (**A**) Anthocyanin accumulation in rosette morphology of WT and transgenic transferred to normal condition (left) and low-Pi stress for 3 days (right). (**B**) Anthocyanin quantification in transgenic lines and WT. (**C**,**D**) Relative expression of anthocyanin biosynthesis genes. LP means low-Pi stress. **, *p* < 0.01; ns, no significance. Scale bar indicates 0.5 cm.

Pi stress significantly inhibits plant growth, and thus plants develop adaptive mechanisms to cope with Pi starvation. He et al. [33] reported that the Pi stress response is closely linked to anthocyanin biosynthesis through the regulation of anthocyanin biosynthesis genes. Therefore, we quantified anthocyanin content in the roots and leaves of transgenic plants under Pi stress treatment. Our results showed no significant difference in anthocyanin content in transgenic and WT plants under normal conditions (Figure 6B). Under Pi stress, however, anthocyanin content in transgenic plants was significantly lower than that in WT (Figure 6B), with a corresponding down-regulation of the anthocyanin biosynthesis genes *AtDFR* and *AtANS* (Figure 6C,D). This suggests that Pi stress response in *ZmARF4* transgenic plants might be inversely related to anthocyanin biosynthesis and that *ZmARF4* is possibly a negative regulator of anthocyanin biosynthesis genes *AtDFR* and *AtANS*

### 2.6. Overexpression ZmARF4 Promoted Plant Growth and Development in Maize

We performed qRT-PCR using RNA isolated from T2 positive transgenic lines in the field to assess the relative expression of *ZmARF4* (Figure S5A). The transcription levels of *ZmARF4* were further validated using Western blot analysis (Figure S4B). Overexpression lines with higher *ZmARF4* expression were selected for phenotypic analysis.

**Figure 7:**
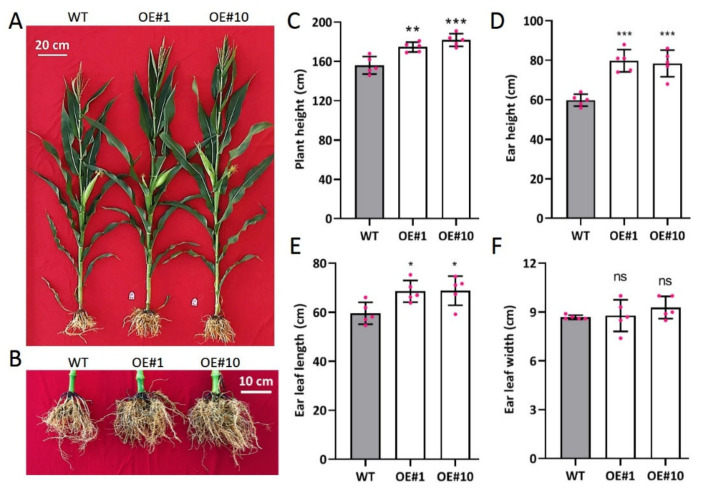
Phenotypic identification of *ZmARF4* overexpressing lines in maize. Plant architecture (**A**) and root system structure (**B**) of WT and *ZmARF4* overexpressing plants (OE#1 and OE#10) were shown in the field. Quantification of agronomic traits with plant height (**C**), ear height (**D**), length of ear leaf (**E**), and width of ear leaf (**F**) in transgenic plants compared to WT. *, *p* < 0.05; **, *p* < 0.01; ***, *p* < 0.001; ns, no significance.

Phenotypic analysis showed that the transgenic plants exhibited significantly stronger phenotypic traits (Figure 7A,B). Except for ear leaf width, transgenic lines recorded significantly higher plant height, ear height, and ear leaf length compared to the WT (Figure 7C–F). The powerful performance of the transgenic line suggests that *ZmARF4* plays a functional role in plant growth and development in maize.

### 2.7. *ZmARF4* Interacted with *ZmILL4*

To elucidate the molecular mechanisms underlying the function of ZmARF4 proteins in initial lateral root regulation and other biological functions, we performed a yeast two-hybrid (Y2H) assay using ZmARF4 as bait to identify the potential interacting proteins. The transcriptional activation activity of ZmARF4 in Y2H Glod was detected using full-length and truncated fragment constructs (Figure S6A). Three classical biochemical functional domains were identified; therefore, we truncated the full-length CDS of *ZmARF4* into three fragments (A, B and C) according to the conserved domains (Figure S6B). All sections containing fragment C (AUX-IAA) could grow on SD-L/-T and SD-L/-T/-H/-A, indicating that AUX-IAA might have functional transcriptional activation properties (Figure S6C).

**Figure 8:**
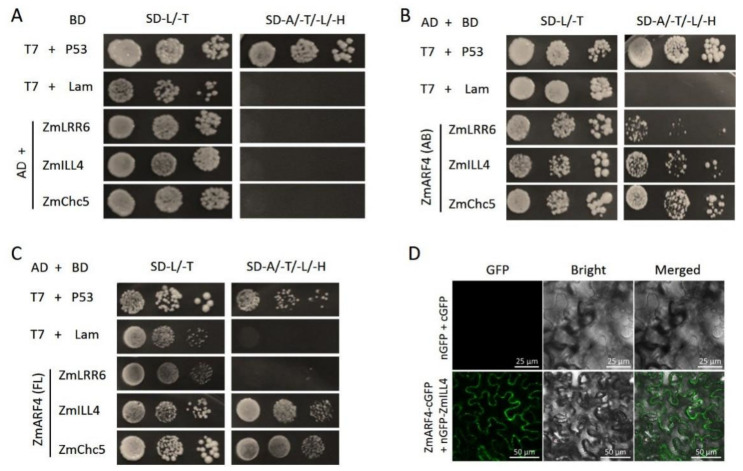
ZmARF4 interacts with ZmILL4 and ZmChc5. (**A**) BD constructs of candidate genes were co-transformed with an empty AD and tested for auto-activation. BD-LRR6, BD-ZmILL4 and BD-ZmChc5 were co-transformed with (**B**) AD-ZmARF4_truncated (AB)_ and (**C**) AD-ZmARF4 (FL, full length) and selected on SD/-Trp/-Leu and SD/-Ade/-Leu/-Trp/-His to confirm interaction. (**D**) The ZmARF4-cGFP and ZmILL4-nGFP fusion genes were co-expressed in the leaves of *N. benthamiana* and GFP fluorescence signal was detected with a confocal microscope.

Consequently, yeast cells harboring a truncated BD-ZmARF4, with no auto-transactional activation potential, were used as bait to screen the prey cDNA library and were selected on SD/-Ade/-Leu/-Trp/-His agar plates. Single colonies were selected, sequenced, and subjected to BLAST analysis. Three candidate genes with the highest hits (ZmLRR6, ZmILL4, and ZmChc5) were selected. To verify the interaction between ZmARF4 selected candidate genes, the coding sequences of these candidate genes were cloned into the BD vector and their transcriptional activation was determined (Figure 8A). The BD constructs of the candidate genes and AD-ZmARF4 were co-transformed into Y2H Gold and selected on SD/-Trp/-Leu and SD/-Ade/-Leu/-Trp/-His. Our results showed that only BD-ZmILL4 and BD-ZmChc5 with Ad-ZmARF4 could grow on SD/-Ade/-Leu/-Trp/-His, indicating that ZmARF4 interacts with ZmILL4 and ZmChc5 in yeast (Figure 8B,C).

The interaction between ZmARF4 and ZmILL4 was further validated using the bimolecular fluorescence complementation (BiFC) assay. The coding sequences of ZmARF4 and ZmILL4 were fused to the C- and N-terminals of a GFP. These constructs, and empty cGFP and nGFP, were transiently co-expressed in *N. benthamiana* leaves in various combinations. Transient co-expression of ZmARF4-cGFP and ZmILL4-nGFP restored GFP fluorescence, which could not be detected in other combinations (Figure 8D).

## 3. Discussion

### 3.1. ZmARF4 Gene Responds to Phosphorus Deficiency Stress

In the long-term evolutionary process, plants have developed an array of adaptations to cope with low-Pi environments through a series of phosphorus starvation responses, including reducing Pi consumption, changing the expression of high-affinity Pi transporters, and changing root architecture [34]. In this study, we investigated the spatiotemporal expression specificity of *ZmARF4* in Pi-tolerant extreme inbred maize lines, combined promoter activity analysis, and applied GUS histochemical staining, and found that *ZmARF4* was mainly induced by phosphorus starvation in roots. In addition, while Pi content in roots did not vary between the genotypes, the relative content of Pi in Arabidopsis leaves overexpressing *ZmARF4* was significantly higher than that in *Col*-0. In particular, after 7 days of low-phosphorus culture, the relative content of Pi in plants decreased compared with normal culture conditions, but the down-regulation of Pi was less pronounced in Arabidopsis overexpressing *ZmARF4* than in *Col*-0 (Figure 5C). In Arabidopsis, the inorganic phosphorus transporter PHT1 family, *PHT1;4*, participates in the transport of inorganic phosphorus and mediates the absorption and transport of Pi in the rhizosphere plant [35,36]. Overexpression of *ZmARF4* significantly increased the expression level of *PHT1;4* in Arabidopsis roots and enhanced the ability of Arabidopsis roots to absorb Pi from the exogenous environment (Figure S7). During phosphorus starvation, plants also recycled inorganic phosphorus in intracellular and extracellular organic phosphorus compounds by up-regulating a series of hydrolases to improve the effective utilization of inorganic phosphorus [37,38]. Our results showed that the expression of nuclease *AtRNS1* in Arabidopsis was up-regulated after low-phosphorus stress, but the up-regulation of the *RNS1* gene in Arabidopsis overexpressing *ZmARF4* was significantly higher than that in *Col*-0 (Figure 5D). It was found that a few purple acid phosphatases with enhanced expression of low phosphorus can be secreted on the root surface and participate in the activation of exogenous organic phosphorus. For example, *AtPAP10* has high ability to hydrolyze adenosine diphosphate (ADP), and overexpression of *AtPAP10* can significantly improve the utilization of ADP in transgenic Arabidopsis [39]. The expression level of the *AtPAP10* gene in Arabidopsis leaves overexpressing *ZmARF4* was significantly higher than that *in Col*-0 (Figure S7). Overexpression of *ZmARF4* improved the ability of plants to utilize ADP and activate exogenous environmental organic phosphorus. These results showed that transgenic Arabidopsis overexpressing *ZmARF4* had a stronger ability to reuse phosphorus and absorb inorganic phosphorus from a low-phosphorus environment than *Col*-0.

Anthocyanin accumulation is the hallmark of plants’ response to low phosphorus [40]. The relative content of anthocyanin in *ZmARF4-*overexpressed plants was significantly lower than that in *Col*-0 plants under low phosphorus, corresponding to the insensitivity of transgenic lines to low phosphorus. Overexpression of *ZmARF4* under low-phosphorus stress significantly reduced the expression of anthocyanin synthesis genes *AtANS* and *AtDFR* in Arabidopsis, which was consistent with the quantification of anthocyanin. Under low-phosphorus conditions, the accumulation of anthocyanin in old leaves decreased and was associated with the high-phosphorus content [41]. Hence, the low anthocyanin content in the leaves of *ZmARF4*-overexpressed plants may be related to the high-phosphorus content in the leaves, while changes in phosphorus content may be associated with phosphorus absorption and translocation.

### 3.2. ZmARF4 Plays a Pivotal Role in Advanced Root Morphogenesis

The lateral root is an important part of plant root morphology, and its formation involves stimulating the proliferation and redifferentiation of matured pericyclic cells [42]. Auxin signals are transmitted through the AUX/IAA-ARFs module, such as IAA14-ARF7/ARF19 and IAA12-ARF5, during the initiation of lateral roots in Arabidopsis [43,44,45,46]. *ARF7* and *ARF19* together with their downstream target genes *LBD16*, *LBD18*, and *LBD29*, form the Auxin-TIR1/ABF2-AUX/IAA-ARF-LBD pathway with auxin, jointly mediate lateral root formation in Arabidopsis [47,48,49]. Our data showed that overexpression of *ZmARF4* partially complemented lateral root emergence and development in the *arf7 arf19* mutant. Under normal conditions, the total root length and root tip number in *ZmARF4*-overexpressed lines were not significantly different from those in *Col*-0, but the root diameter and root volume were significantly higher than those in *Col*-0. Under low-phosphorus stress, the normal growth of Arabidopsis was inhibited, with a reduction in primary root length and an increase in lateral root growth. However, the total root length, root surface area, root volume, and root tip number of Arabidopsis *thaliana* overexpressing *ZmARF4* were significantly higher than those of *Col*-0. Zhang et al. found that *PRH1*, a downstream gene of *ARF7* and *LBDs*, is involved in the regulation of auxin-induced lateral root development. Overexpression of *PRH1* did not produce any phenotype related to lateral roots in WT, yet partially restored the phenotype of lateral roots in *arf7*, *lbd16*, *1bd18*, and *lbd29* [50]. These results are similar to those of our study.

ARFs members in plants always exhibit functional redundancy or antagonism. In our previous investigation (unpublished data), the evolution analysis of *ARFs* in maize, rice, and Arabidopsis showed the ARFs family was divide into branches. The *ZmARF4* focused on in this article was classified as belonging to the homologous with *OsARF11* and *AtARF5*. The classical auxin response pathway mediated by *ARF5/MONOPTEROS (MP)* plays an important role in the regulation of radicle specialization, vascular tissue development, and shoot tip development in Arabidopsis [51,52,53,54]. In addition, the *OsARF1*1 plays a central role in supervising the formation of lateral roots, panicle branches, and grain meristem and is also involved in leaf vein and other organ growth [55,56]. The initial occurrence of lateral roots is from pericycle cells located in the outermost layer of the root pericycle. After the formation of pericycle cells, they maintain the ability of cell division for a long time, so that plants can form lateral roots flexibly in response to environmental changes, which has been proven to be related to auxin-mediated transcriptional regulation [47]. ARF7 and ARF19 were found to be strongly expressed at the beginning of lateral root development in Arabidopsis [57]. We detected GUS expression in the pericycle of the primary root mature zone in Arabidopsis, but no signal was detected in the lateral root (Figure S3). We speculated that ZmARF4 may play a role in the initiation of the early stage of lateral root emergence. Our results suggest that ZmARF4 may have a biological function similar to AtARF7/AtARF19; that is, it participates in lateral root initiation and affects root morphogenesis. In addition, an interacting protein, ZmILL4, was screened using a Y2H library. ZmILL4 is an IAA amino acid hydrolase that regulates the rate of hydrolysis of amido-IAA in the endoplasmic reticulum of Arabidopsis and activates auxin signaling [58]. Y2H rotation verification and BiFC experiments showed that ZmARF4 interacted with ZmILL4. Therefore, we speculate that *ZmARF4* may also indirectly regulate the occurrence of lateral roots by participating in the synthesis, transportation, or hydrolysis of auxin, but the specific mechanism remains to be determined.

### 3.3. ZmARF4 Is Involved in the Defense Response Pathway in Maize

At present, studies on the function of the *ARF* gene have mainly focused on the regulation of plant growth and development, while a few studies have reported the participation of ARFs in defense responses in plants. Several different plant RNA viruses weaken their mediated antiviral defense response by targeting the function of *OsARF17*, which is conducive to virus infection [59]. The functional loss mutants of *osarf12* or *osarf16* showed decreased resistance to RDV, while *osarf11* or *osarf5* showed increased resistance to RDV [60]. We screened some proteins that may interact with ZmARF4 through a Y2H. Most of the gene functional annotations are mainly related to plant growth, development, and defense responses, such as ZmLRR6 and ZmChc5. ZmLRR6 is a stretch-like protein with the PLN00113 superfamily domain, which is rich in leucine. The first documented plant LRR proteins are serine/threonine kinase receptors and polygalacturonase inhibitors, which are involved in plant defense responses and developmental processes [61].

The cell wall is an important part of the plant defense response and provides protection against both biological and abiotic stresses. Baumberger et al. found that identified Arabidopsis LRX1 is as a cell wall component involved in regulating cell expansion [62]. Many studies have shown that the LRX protein LRR domain is the binding site of RALF with high affinity [63,64,65,66]. RALFs are peptide hormones that can regulate plant growth [67] and pathogen infection by inducing various physiological responses [68]. ZmChc5 is a chitinase that contains a GH18_hevamine_XipI_class_III conserved domain. This domain family includes xylanase inhibitors XIP-I and plant chitinases III, which play an important role in the defense against pathogenic bacteria and fungi. Chitinase acts on the cell wall of fungi, degrades the chitin components in the cell wall, and destroys the cytoskeleton of fungi, thus inhibiting the pathogenicity and growth of fungi, and achieving the antifungal effect [69,70,71]. Gao and Zhao explored a chitinase-mediated fungal defense model using model plants. In *Brassica juncea*, BjMYB1 enters the nucleus and binds to Wbl4 in promoter BJC-P to activate the expression of BjCHI1. Accumulated BjCHI1 contributes to the cleavage of chitin in fungal cell walls, thereby defending against fungal infection [72]. Our results show that ZmARF4 can interact with ZmLRR6, ZmChc5, and other proteins in the yeast system, suggesting that ZmARF4 may play an important role in the plant defense response pathway, but the specific mechanism of action remains unclear.

## 4. Materials and Methods

### 4.1. Plant Materials and Plant Transformation

All *Arabidopsis thaliana* plants used in this study were from the *Col*-0 background. The double mutant *arf7 arf19* lost the initiation of lateral roots [73]. To generate overexpression lines of Arabidopsis, the coding sequence of *ZmARF4* was amplified and cloned into the *Bam* HI and *Xba* I sites of the CPB expression vector under the control of a CaMV 35S promoter. The *ZmARF4* promoter was also amplified and cloned into the *Nco* I and *Hin* dIII sites of pCAMBIA3301 to replace the CaMV 35S promoter upstream of the *GUS* gene. An approximate 2-kb genomic DNA fragment of the *ZmARF4* promoter upstream of the start codon was amplified from the purified DNA of both Pi-tolerant 178 and Pi-sensitive 9782 lines. All fragments were ligated to the lined vector by recombination using the ClonExpress^®^ II system (Vazyme Biotech, Nanjing, China). The constructs were transformed into *Agrobacterium* strain EH105. The WT strain was transformed with pCAMBIA3301-Pro*_ZmARF4_*::*GUS* and CPB-Pro_35S_::*ZmARF4*. The *arf7 arf19* mutant was transformed with CPB-Pro_35S_:*ZmARF4*. Positive pCAMBIA3301-Pro*_ZmARF4_*::*GUS* and CPB-Pro_35S_:*ZmARF4* were screened on half-strength MS supplemented with 50 μg/mL of glufosinate–ammonium. Homozygous lines in the T4 generation with high expression of *ZmARF4* were selected for subsequent experiments.

To generate maize overexpression lines, the open reading frame of *ZmARF4* was cloned into the CUB expression vector at the BamHI and SacI sites. The constructed plasmid of CUB-Pro*_UBI_*::*ZmARF4-*3×Flag was sent to Wimi Biotechnology for maize transformation. Wild line KN5585 was used as the transformation receptor. T2 lines were planted in Hainan and screened using Basta. Positive lines with higher expression levels were selected for subsequent phenotypic evaluation.

### 4.2. Plant Growth Condition and Stress Treatment

Half-strength MS medium with 1.5% (*w*/*v*) sucrose and 0.6% (*w*/*v*) agar powder (Solarbio Cat No. A8190) was used as the standard Pi sufficient medium (PA). Half-strength MS without phosphate (Caisson Lab. REF: MSP11-10LT) and with 1.5% (*w*/*v*) sucrose and 0.6% (*w*/*v*) agar powder (Solarbio Cat No. A8190) was supplemented with PA (100:1) to represent low-Pi medium (LP). Half MS strength medium supplemented with 80 mM NaCl and 200 mM mannitol was used to induce salinity and osmotic stress, respectively.

The WT, *arf7 arf19*, and transgenic lines of Arabidopsis were surface-sterilized in 75% ethanol and 5% NaOCl and was rinsed five times with sterilized distilled water. Seeds were placed in the PA medium and kept upright at 4 °C for 3 nights. Plates were then transferred to a growth chamber and kept upright at 23 °C and a 16-h light/8-h dark cycle for 7 days. Uniform seedlings of the WT and transgenic lines were transferred to PA and stress induction media plates to trigger stress responses. Seedlings were evaluated for root phenotypic traits and the quantification of Pi and anthocyanin content.

### 4.3. Root Phenotypic Traits in Arabidopsis

The WT, *arf7 arf19*, and transgenic lines were subjected to phenotypic evaluation after stress imposition. The WinRhizo Pro 2008a image analysis system (Regent Instruments Inc., Quebec City, Canada) was used to analyze root morphological traits, including total root length, total root surface area, average root diameter, total root volume, and total root tips under normal and stress conditions. The data obtained were subjected to analysis of variance in SPSS to test for statistical significance between the lines.

### 4.4. Measurement of Pi and Anthocyanin Contents

Pi was estimated in the roots and leaves of WT and transgenic plant materials after 7 days of low-phosphate treatment by a modification of the protocol described by Nanamori (2004). Approximately 0.1 g of each sample was weighed into 2-mL Eppendorf (EP) tubes and ground to a fine powder in liquid nitrogen. The samples were then homogenized in an extraction buffer (10 mM Tris-HCl, 1 mM EDTA, 10 mM NaCl, 1 mM Beta-ME, and I mM PMSF; pH 8.0) and incubated at room temperature for 30 min. Approximately 100 μL of the homogenate was separated into new 1.5-mL EP tubes and 900 μL of 1% acetic acid was added to each tube and mixed thoroughly. After centrifugation at 16,000× *g* for 5 min, 300 μL of supernatant was separated into new 1.5-mL EP tubes. Approximately 700 μL of the analysis buffer (0.35% (NH_4_)_2_.MoO_4_, 0.86N H_2_SO_4_, and 1.4% Vitamin C) was added to each sample and kept at 42 °C for 30 min. The phosphate content was determined by measuring the absorbance at A_820_. Phosphate concentration was determined by normalization of fresh weight, and the values obtained were subjected to statistical analysis to determine significant differences between the samples.

Anthocyanin content was quantified in the roots and leaves of WT and transgenic plant materials after 3 days of low-phosphate treatment, following the procedure described by Gou et al. [74]. Approximately 0.1 g of each tissue was weighed into 2-mL EP tubes and ground into a fine powder using liquid nitrogen.

The samples were then homogenized in 300 μL of 0.1% HCl-methanol and incubated overnight at room temperature. Anthocyanin was separated from chlorophyll by the addition of 200 μL of distilled water and 500 μL of chloroform, followed by short vortexing and spinning. Anthocyanin quantification in the aqueous phase was performed using a spectrophotometer at the absorbance of A_530_ and A_657_. The anthocyanin content in each sample was calculated by subtracting A_657_ from A_530_.

### 4.5. GUS Activity Detection

The *Agrobacterium* strain GV3101 harboring the Pro*_ZmARF4_*::*GUS* construct was transiently expressed in tobacco leaves. Subcellular GUS activity detection was carried out after 36 h using a microscope. GUS activity was further detected in transgenic plants harboring the Pro*_ZmARF4_*::*GUS* construct. WT and transgenic seedlings were grown for 7 days on 1/2 MS medium plates, as described above. Uniform seedlings were transferred to PA and LP mediums for 3 days, and then GUS activity in the seedlings was detected. Seedlings were incubated overnight at 37 °C in GUS staining buffer (2 mM 5-bromo-4-chloro-3-indolyl- β-D-glucuronic acid in 50 mM sodium Pi buffer, pH 7.2) containing 0.1% Triton X-100, 2 mM K_4_Fe(CN)_6_, 2 mM K_3_Fe(CN)_6_, and 10 mM EDTA. The stained seedlings were transferred to 100% (*v*/*v*) ethanol for 1 h to remove chlorophyll. GUS activity was then detected using a light microscope (Olympus IX73).

### 4.6. RNA Extraction and Quantitative Real Time-Polymerase Chain Reaction

Two low-Pi-sensitivity extreme maize inbred lines P178 and 9782 were treated for 0, 4, 12, 24, 36, and 48 h with low-phosphorus stress at the seedling stage. In particular, samples were collected at the same time after different treatment times. The culture conditions of the materials were similar to those used in our previous studies [75]. Total RNA was extracted from plant materials using TRIzol reagent (Invitrogen, Carlsbad, CA, USA). First-strand cDNA was synthesized using *TransScript*^®^II One-Step gDNA Removal and cDNA Synthesis SuperMix (TransGen, Beijing, China). The cDNA was diluted five-fold with nuclease-free water and used as a template for quantitative reverse-transcription polymerase chain reaction (qRT-PCR) analysis. qRT-PCR was performed in three technical replicates using *TransScript*^®^II Green One-Step qRT-PCR SuperMix (TransGen, Beijing, China). The expression of the housekeeping *ZmUBI1* gene was used in maize and *IPP2* in Arabidopsis was used as an internal control. The primers used are shown in Table S1.

### 4.7. Western Blot Analysis

Western blot analysis was carried out to quantify the accumulation of ZmARF4 protein in the respective plant materials. Total protein from maize leaf tissues was extracted using cell lysis buffer (50 mM Tris-HCl pH 7.5, 150 mM NaCl, 0.2% NP40, 0.1% Triton X-100 and 1 mM phenylmethylsulfonyl fluoride (PMSF)). Total protein from Arabidopsis was extracted using 10% NP-40 solution (Coolaber Cat No. SL9320-100) and 1 mM PMSF. Protein extracts with 5× sodium dodecyl sulfate (SDS) loading buffer were separated on 10% SDS-polyacrylamide gel, transferred to a polyvinylidene difluoride membrane, and subjected to Western blotting analysis using anti-Flag and anti-GUS monoclonal primary antibodies in maize and Arabidopsis, respectively. Secondary horseradish peroxidase-conjugated goat anti-rabbit antibody was used at a dilution ratio of 1:2000. Protein visualization was performed using an enhanced chemiluminescence kit.

### 4.8. Subcellular Localization

The coding sequence of *ZmARF4* was amplified and fused to the pCAMBIA2300 expression vector at the BamHI and SpeI sites, driven by the CaMV 35S promoter. The gene-specific primers used are listed in Table S1. The Pro_35S_:*ZmARF4*-eGFP construct was transformed into maize mesophyll protoplasts by the polyethylene glycol-mediated method, as outlined by Liu et al. [76], and cultured overnight in darkness at 22 °C. Subcellular detection of green fluorescence protein (GFP) expression was carried out using a confocal laser scanning microscope (Olympus IX73).

### 4.9. Yeast Two-Hybrid cDNA Library Screening and Confirmation

The CloneMiner^TM^ II cDNA library construction kit (Invitrogen, Carlsbad, CA, USA) was used to construct a cDNA library in P178 maize seedlings grown under neutral day-length conditions (12-h light/12-h dark) in modified Hoagland solution with low Pi for 10 days. High-quality cDNA libraries were constructed into pGADT7 (AD) vector and transformed into Y187 competent yeast cells by OEbiotech (Shanghai, China). The Y2H library screening was performed using the Clontech two-hybrid system according to the manufacturer’s instructions. The constructed carrier, Y2HGold competent yeast cells with pGBKT7-ZmARF4 (BD-ZmARF4), was used to screen the P178 cDNA library after it was tested for auto-activation as a bait vector. The transformants were screened on SD/-Ade/-Leu/-Trp/-His agar plates and incubated for 2–4 days at 28 °C. Prey plasmids were extracted and sequenced from single blue colonies, which were putatively positive clones.

To further confirm the interactions, candidate genes from positive clones were inserted into BD vectors and their interaction abilities were verified by co-transformation with AD- ZmARF4 into Y2HGold strains. pGBKT7-53 and pGBKT7-Lam were co-transformed with pGADT7-T as positive and negative controls, respectively. Transformants were plated and cultured on SD/-Trp/-Leu and SD/-Ade/-Leu/-Trp/-His agar plates to test for interactions.

### 4.10. Bimolecular Fluorescence Complementation Assay

We performed BiFC to validate the interaction between ZmARF4 and ZmILL4. The coding sequences of *ZmARF4* and *ZmILL4* were amplified and cloned into the binary vector pXY104-cGFP and pXY106-nGFP to obtain ZmARF4-cGFP and ZmILL4-nGFP constructs driven by the CaMV35S promoter. These constructs, together with empty cGFP and nGFP, were used to transform the *Agrobacterium* strain GV3101. Various combinations of these constructs were co-expressed in the leaves of *N. benthamiana*. The GFP fluorescence signal was detected using a confocal microscope.

## Supplementary Materials

The following supporting information can be downloaded at: https://www.mdpi.com/article/10.3390/ijms23042361/s1.
