# Supplementary material for: Maize Transcription Factor ZmARF4 Confers Phosphorus Tolerance by Promoting Root Morphological Development
Source: Int J Mol Sci. 2022 Feb 21;23(4):2361. doi: 10.3390/ijms23042361 (PMC8880536; doi:10.3390/ijms23042361)
Supplement: Supplementary file 1 [file ijms-23-02361-s001.zip › ijms-1524944-supplementary.pdf]

Supplementary material

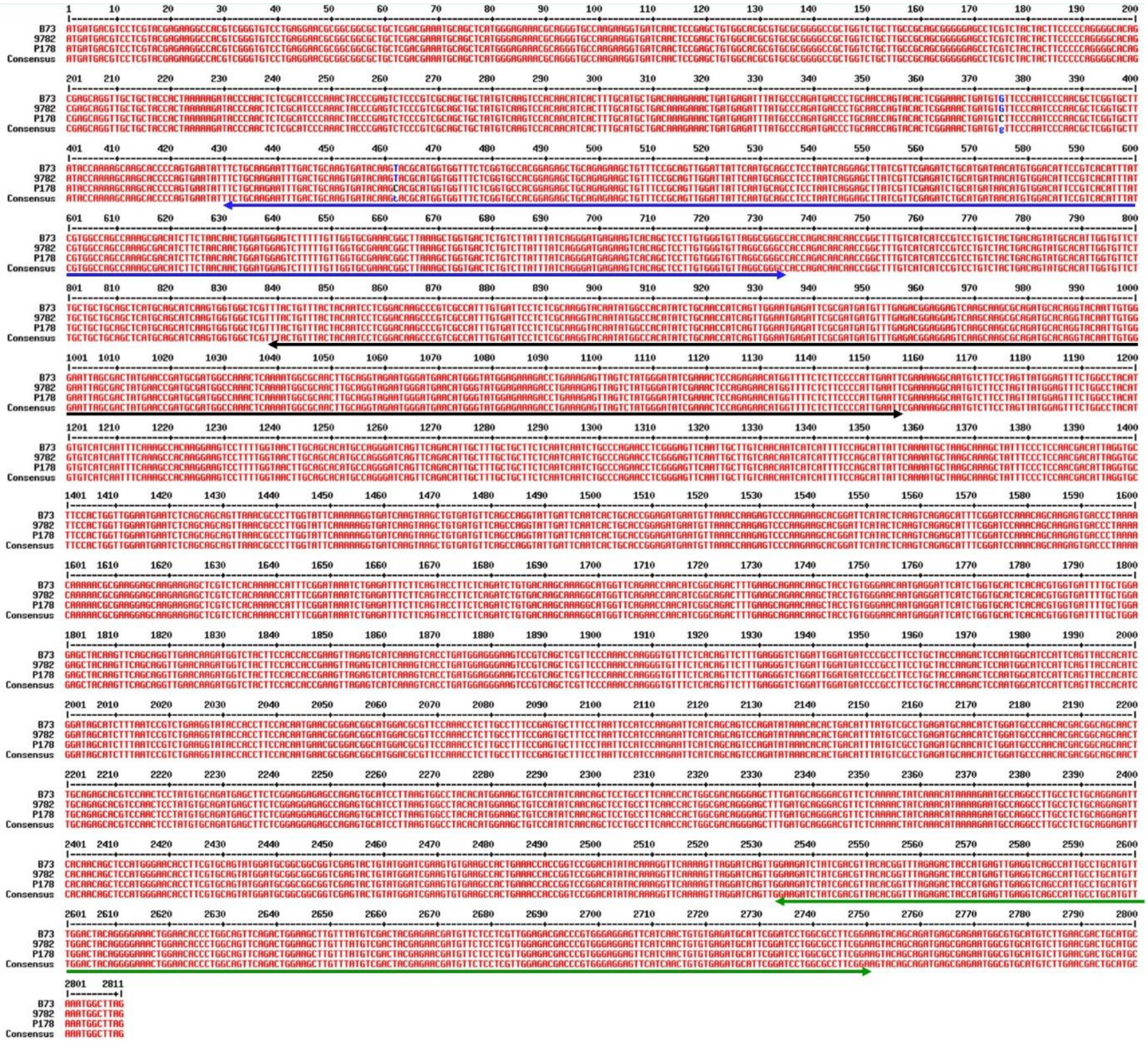

Figure S1. Alignment of *ZmARF4* coding sequence from Pi-tolerant (P178) and -sensitive (9782) lines of maize. Only two identified single-nucleotide polymorphisms were synonymous mutations in the coding sequence, suggesting that the coding sequence is highly conserved. ZmARF4 contains three domains, as B3 domain shown with blue arrow (430 – 735bp), Auxin response domain shown with black arrow (838–1056bp) and AUX _ IAA super family shown with green arrow (2533 – 2751bp).

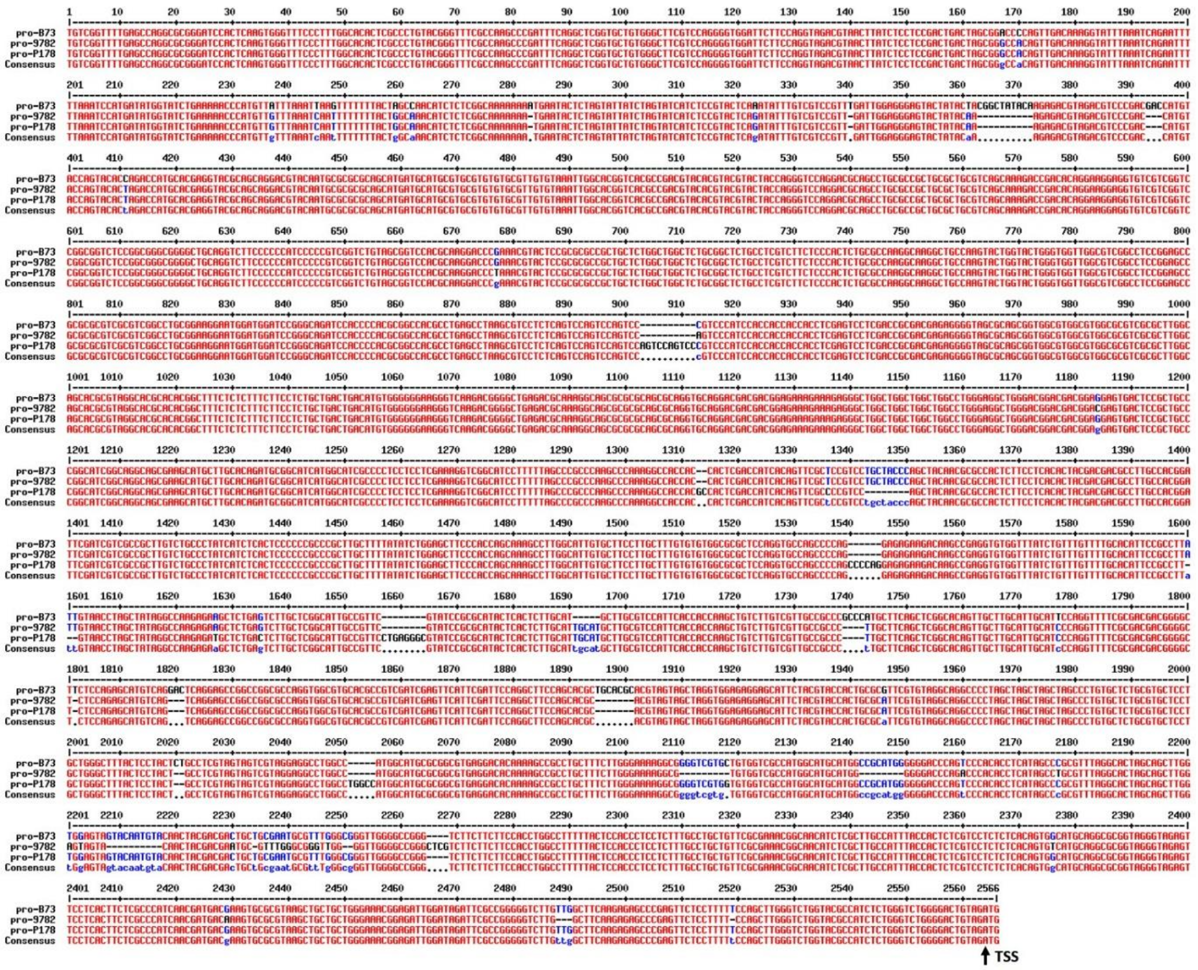

Figure S2. Alignment of promoter sequence of *ZmARF4* from Pi-tolerant (P178) and -sensitive (9782) lines of maize. Several sequence polymorphisms were identified and could serve as useful alleles for trait improvement in maize. Arrow indicates translation start sites (TSS).

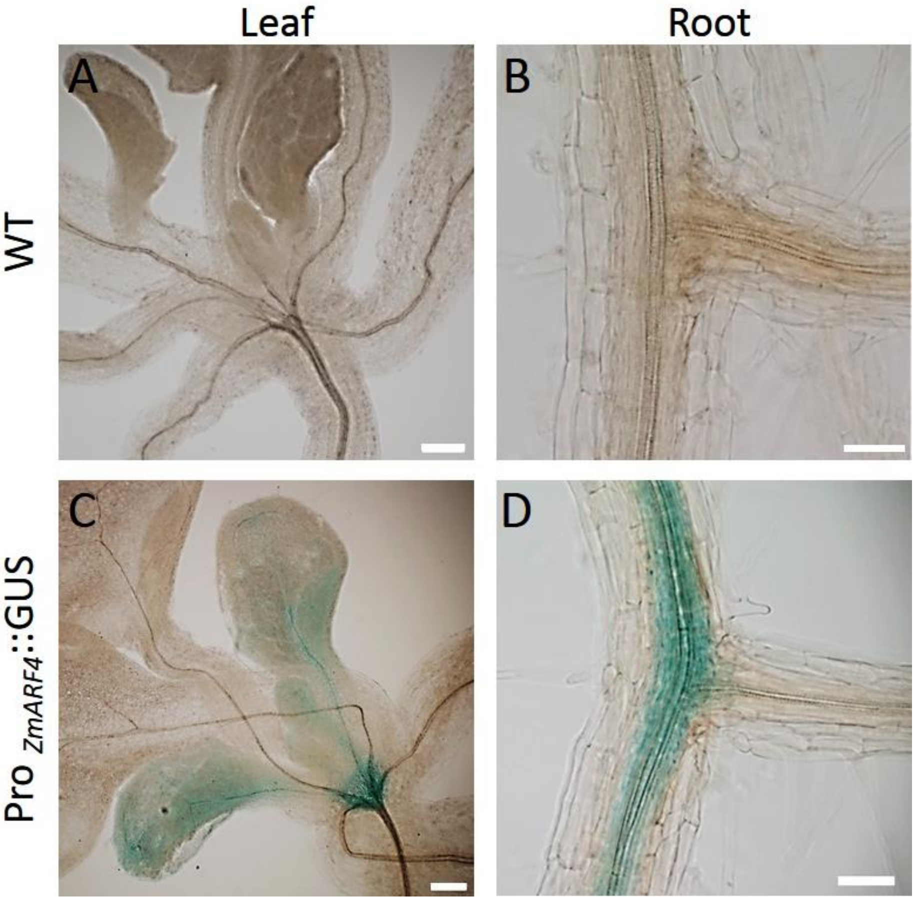

Figure S3. Histochemical staining of Pro$_{ZmARF4\text{-P178}}$::*GUS* detection. GUS activity in transgenic lines of Arabidopsis was detected in leaves and the stele of roots. *Arabidopsis thaliana* ecotype *Columbia*-0 was used as the receptor material in this study.

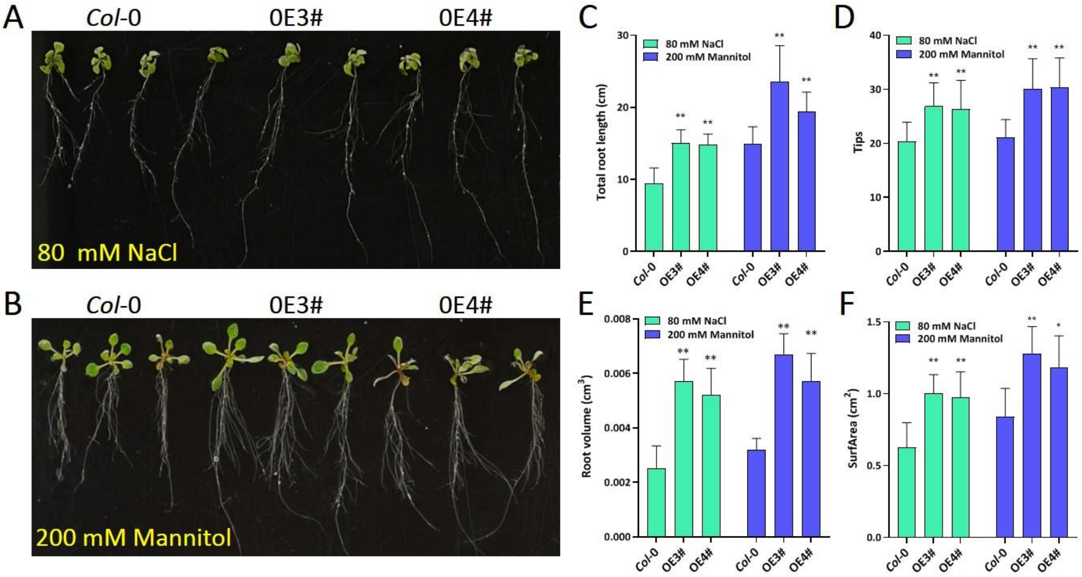

Figure S4. *ZmARF4* improved the tolerance of other abiotic stresses in Arabidopsis. Seven-day old seedlings were transferred to ½ MS plates supplemented with (A) 80 mM NaCl and (B) 200 mM mannitol to induce salinity and osmotic stress, respectively. Transgenic plants displayed better root phenotypic characteristics of (C) total root length, (D) root tips, (E) root volume, and (F) root surface area than the wild type. *, P<0.05; **, P<0.01.

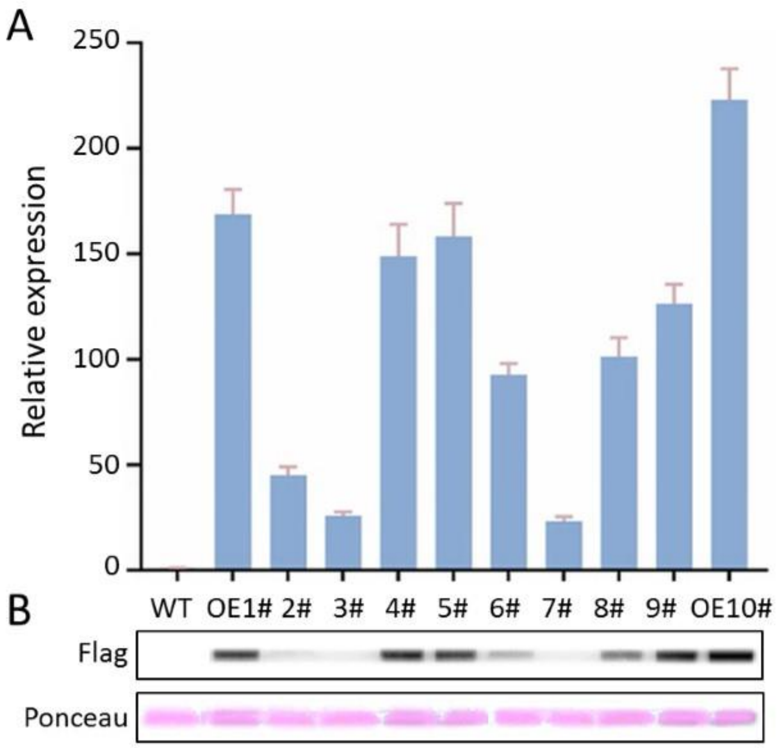

Figure S5. Detection of *ZmARF4* overexpression transgenic lines of maize. (A) Transcript levels of *ZmARF4* were determined among transgenic maize lines. (B) Expression levels were validated with western blotting analysis.

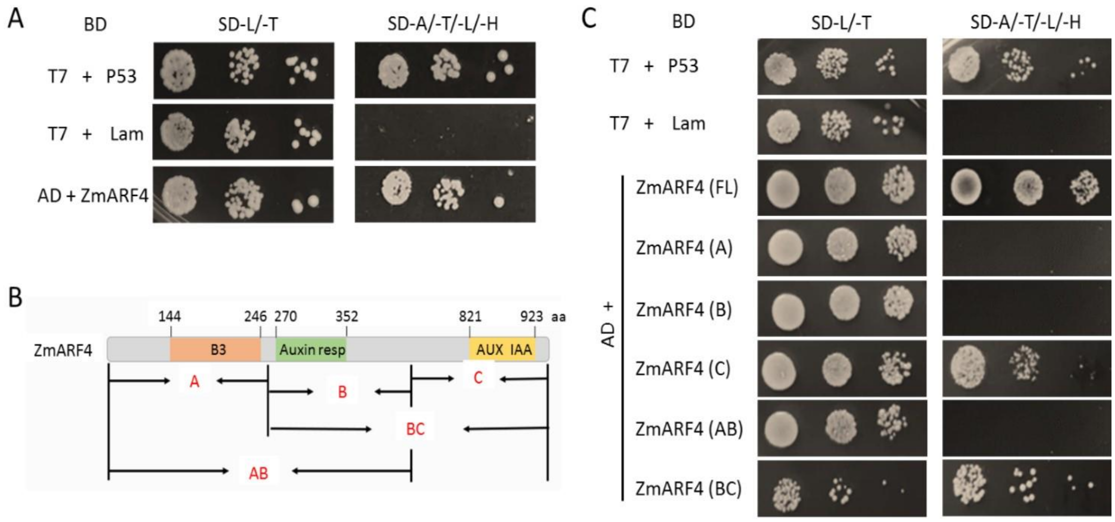

Figure S6. Determination of auto-activation activity of ZmARF4. (A) BD-ZmARF4 was co-transformed with empty AD and selected on SD/-Trp/-Leu and SD/-Ade/-Leu/-Trp/-His. (B) Identification of the conserved domains of the ZmARF4 protein. (C) The AUX/IAA domain was responsible for the auto-activation activity of the ZmARF4 protein. pGBKT7-53 and pGBKT7-Lam were co-transformed with pGADT7-T as positive and negative controls, respectively. FL, full length.

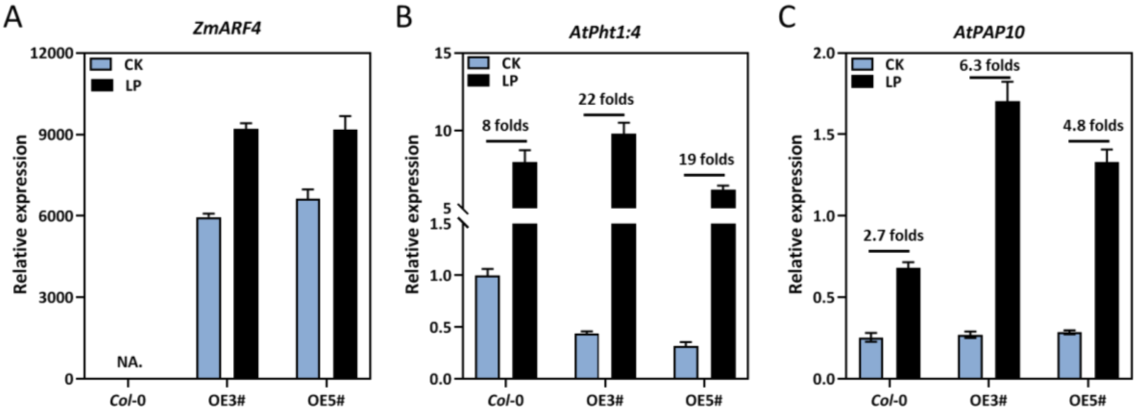

Figure S7. Expression of phosphorus response related genes in root of *ZmARF4* transgenic lines in Arabidopsis. CK is 1/2MS; LP is treated in low Pi (1 μM).

Table S1. All primers used in this study.

Prime name	Sequence(5’-3’)
pARF4-3301GUS F	GACCTGCAGGCATGCAAGCTTTGTCGGTTTTGAGCCAGGCG
pARF4-3301GUS R	TTACCCTCAGATCTACCATGGCTACAGTCCCCAGACCCAGA
2300-ZmARF4F	ATTTGGAGAGGACAGGGTACCATGATGACGTCCTCGTACGA
2300-ZmARF4R	GGTACTAGTGTCGACTCTAGAAGCCATTTGCATGCAGTCGT
AD-ZmARF4F	GCCATGGAGGCCAGTGAATTCATGATGACGTCCTCGTACGA
AD-ZmARF4FR	ACGATTCATCTGCAGCTCGAGCTAAGCCATTTGCATGCAG
AD-ZmARF4(AB)F	GCCATGGAGGCCAGTGAATTCATGATGACGTCCTCGTACGAG
AD-ZmARF4(AB)R	ACGATTCATCTGCAGCTCGAGAACCTGCTGAACTTGTAGCTCT
BD-ZmARF4F	ATGGCCATGGAGGCCGAATTCATGATGACGTCCTCGTACGA
BD-ZmARF4R	CTAGTTATGCGGCCGCTGCAGCTAAGCCATTTGCATGCAG
BD-ZmARF4AF	ATGGCCATGGAGGCCGAATTCATGATGACGTCCTCGTACGAG
BD-ZmARF4AR	CTAGTTATGCGGCCGCTGCAGTGATGACAAAGCCGGTTGTTG
BD-ZmARF4BF	ATGGCCATGGAGGCCGAATTCTCCGTCCTGTCTACTGACAGT
BD-ZmARF4BR	CTAGTTATGCGGCCGCTGCAGAACCTGCTGAACTTGTAGCTCT
BD-ZmARF4CF	ATGGCCATGGAGGCCGAATTCGAACAAGATGGTCTACTTCCAC
BD-ZmARF4CR	CTAGTTATGCGGCCGCTGCAGCTAAGCCATTTGCATGCAGTCG
BD-ZmLRR6F	ATGGCCATGGAGGCCGAATTCATGAAGAAGGAGGAGGTGGCC
BD-ZmLRR6R	CTAGTTATGCGGCCGCTGCAGCTAGTAGTGCGGCGGCGGC
BD-ZmILL4F	ATGGCCATGGAGGCCGAATTCATGGGCGCGCCCGCGCCGAG
BD-ZmILL4R	CTAGTTATGCGGCCGCTGCAGCTAAAGCTCGTCGTGGGGACC
BD-ZmILL4F	ATGGCCATGGAGGCCGAATTCATGGCGGCTAATCTCAAGTGGGC
BD-ZmILL4R	CTAGTTATGCGGCCGCTGCAGCGACGCTGCCCTTCACCTGGCT
qGUSF	CGGTCAGTGGCAGTGAAGGG
qGUSR	CGAGGTACGGTAGGAGTTGG
qAtACT2F	GGAAGGATCTGTACGGTAAC
qAtACT2R	TGTGAACGATTCCTGGACCT
qZmACTF	TACGCTAGTGGGCGAACAAC
qZmACTR	CATTAGGTGGTCGGTGAGGT
qZmGAPDHF	CCATCACTGCCACCCAGAAAAC
qZmGAPDHR	AGGAACACGGAAGGACATACCAG
qAtDFRF	TGCCATAAACGGATGTGACG
qAtDFRR	ACATTCCATTCACTGTCGGCT
qAtANSF	GGCTGTGTTTTGTGAGCCACCA
qAtANSR	CCTTGGAGGAAACTTAGCCGGAGA
qAtRNS1F	TGATGCCTCTAAACCATTCGAT
qAtRNS1R	TACCATGCTTCTCCCATTCG
qAtPAP10F	AGACACTCAACGAGGACTCAC
qAtPAP10R	CCGCCATTAAACCCTTCCACA
qAtPHT1;4F	ACCCAATGCTACAACCTTCG
qAtPHT1;4R	CTGGGTTCTGAGCCAAGTAC
